# 94 Effects of Palliative Care Consultation Triggers in the Burn Unit

**DOI:** 10.1093/jbcr/irae036.093

**Published:** 2024-04-17

**Authors:** Alisa Savetamal, Curtis Swanson

**Affiliations:** Connecticut Burn Center, Bridgeport Hospital, Bridgeport, Connecticut; Connecticut Burn Center, Bridgeport Hospital, Bridgeport, Connecticut

## Abstract

**Introduction:**

Palliative care consultation has become increasingly utilized as an important part of the care of severely ill and chronically ill patients. In our burn center, in collaboration with our palliative care specialists, we introduced automatic "triggers" for palliative care consultation. We seek to assess how implementation of these triggers has changed the hospital courses of patients who died in the burn unit of their injuries. Our burn center has previously investigated the characteristics of patients succumbing to their burn injuries. In this review, we here compare our current results to the pre-trigger era.

**Methods:**

A pre/post, retrospective review of all adult patients who died in the burn center between 2020 and 2023 was undertaken and compared to results of our previous study (2013-2017). Patient demographics were obtained (age; gender; TBSA; presence of inhalation injury; LOS; Baux scores). All patients in the later era received a palliative care (PC) consultation. Patients without burn injury were excluded (complex wounds; SJS/TENS). Patients in both time periods were stratified into early, intermediate, and late death groups ( < 48 hours; >48 hours and < 14 days; >14 days).

**Results:**

From January 2020 to June 2023, twenty patients were identified who died of burn injuries. Of these, six (30%) were female). The average age of all of the patients was 59.4, average TBSA was 50.4, and average Baux scores of all patients was 112.4. Patients were stratified into early, intermediate, and late groups (3, 11, and 6 patients respectively; the previous review had 16, 13, and 13). The average Baux scores after stratification were 132.3, 125, and 99.5, similar to previous findings of 133.6, 93.7, and 102.6 (p>0.05). The average number of procedures in each group was 0.3, 2.5, and 3.3, compared to previous findings of 0, 1.1, and 4.6 (p = 0.35). Average length of stay in the "late" group was 25.3 days, compared to 31.7 days in the previous review (p=0.48).

**Conclusions:**

After implementation of palliative care triggers in our burn center, a greater proportion of patients fell into the intermediate death group (55% vs 30.9% previously), suggesting that having earlier palliative care conversations helped to guide families and patients in making difficult but important end-of-life discussions. In addition, the number of procedures per patient was reduced in the late death group after the institution of palliative care triggers. Length of stay in the "late" group, which was the only group not time-limited by stratification, decreased by an average of 6 day. While these findings were not statistically significant. they do suggest that PC consultation helped to guide the team and patients' families in surgical decision-making in this group of largely older patients.

**Applicability of Research to Practice:**

Palliative care consultation can be of benefit and should be considered early for burn patients meeting institutional criteria.

